# Inhibition of BRD4 inhibits proliferation and promotes apoptosis of psoriatic keratinocytes

**DOI:** 10.1186/s12938-021-00943-y

**Published:** 2021-10-21

**Authors:** Xiaohui Sun, Pengfei Yang

**Affiliations:** 1 Department of Dermatology, Shandong Provincial Qianfoshan Hospital, Cheeloo College of Medicine, Shandong University, Jinan, 250014 Shandong China; 2 Department of Dermatology, The First Affiliated Hospital of Shandong First Medical University & Shandong Provincial Qianfoshan Hospital, Jinan Clinical Research Center for Tissue Engineering Skin Regeneration and Wound Repair, 16766 Jingshi Road, Jinan, 250014 Shandong China

**Keywords:** BRD4, Proliferation, Apoptosis, Psoriatic keratinocytes

## Abstract

**Background:**

Psoriasis is a common chronic recurrent inflammatory skin disease. The pathogenesis of psoriasis, such as other autoimmune diseases, is still unclear, which brings great difficulties to the treatment. This study aimed to investigate the role of bromine domain protein 4 (BRD4) in affecting the psoriatic keratinocytes.

**Methods:**

Imiquimod-induced psoriasis mice model and TNF-α or IL-17A induced HaCAT cells, an experimental model in vitro for psoriasis, were constructed. The pathological skin changes at the back of mice were observed by hematoxylin and eosin (H&E) assay and evaluated by psoriasis area and severity index (PASI). KI67 expression and keratinocyte apoptosis at the skin tissues were, respectively, detected by Immunohistochemical analysis and TUNEL assay. The inflammatory factors in mice serum and culture supernatant were determined by ELISA assay. The related proteins expression of proliferation, apoptosis and MAPK pathway were detected by Western blot analysis.

**Results:**

BRD4 expression was upregulated in injured skin on the back of imiquimod-induced mice and (+)-JQ1 relieved the skin injury by suppressing the inflammation and promoting apoptosis of keratinocytes. Consistently, BRD4 expression was also increased in TNF-α or IL-17A induced HaCAT cells. (+)-JQ1 suppressed the viability and inflammation, and promoted apoptosis of TNF-α or IL-17A induced HaCAT cells. In addition, the MAPK signaling pathway was inhibited by (+)-JQ1 whether in mice or HaCAT cells.

**Conclusions:**

Inhibition of BRD4 inhibited proliferation and inflammation and promoted apoptosis of psoriatic keratinocytes.

## Background

Psoriasis is a chronic inflammatory skin disease. About 2% of adults worldwide suffer from psoriasis, which can cause itching, silvery scales and swollen erythema, and can lead to complications of depression. Psoriasis is also associated with many diseases, such as hypertension, diabetes and metabolic syndrome [1–3]. Physiologically, the manifestations of psoriasis vulgaris include epidermal hyperkeratosis and paraceratosis, vascular distortion, and dermal inflammatory infiltration [4]. However, the pathogenesis of psoriasis is still unclear. Current systematic treatment measures for psoriasis include methotrexate (MTX), cyclosporin A, auxin, biologics, and sometimes steroids for patients with moderate to severe psoriasis, but these drugs have obvious side effects and adverse reactions, such as bone marrow suppression, abnormal liver function and metabolic disorders [5]. Therefore, it is urgent to explore new effective therapeutic drugs for psoriasis.

Bromine domain protein 4 (BRD4) is the most widely studied member of the bromodomain and extra-terminal (BET) family, which contains two tandem bromine domains (BD1, BD2) and one extra-terminal domain (ET) [6]. BRD4 induces activation or inhibition of target genes by recruiting different transcriptional regulators throughout the cell cycle [7] and, meanwhile, regulates DNA replication, cell cycle gene transcription and other cell activities [8]. A study has shown that BRD4 has pro-inflammatory effects [9]. In cerebral ischemia–reperfusion injury, BRD4 inhibition can reduce glial cell activation and inhibit inflammatory release [10]. In the spinal cord injury model, BRD4 inhibition attenuates the inflammatory response of microglia [11]. BRD4 inhibition can alleviate the vincristine-induced peripheral neuropathy by suppressing inflammation and oxidative stress [12]. However, there are few studies on BRD4 in psoriasis which is a chronic inflammatory disease. Only one study has shown that miR-125 can inhibit the expression of BRD4 through Notch signaling in psoriasis [13]. However, the specific effect of BRD4 inhibition on psoriasis is still unknown.

Studies have shown that inhibition of BRD4 can inhibit MAPK signal and reduce spinal cord ischemia reperfusion injury [11] and inhibition of BRD4 can inhibit the expression of MAPK signaling pathway in diabetic intervertebral disc degeneration [14]. Therefore, this study aims to continue to explore whether BRD4 inhibition can affect MAPK signaling pathway in psoriasis. In addition to an animal model, a cell model also used in this study can be more fully verify the regulatory effect of BRD4 inhibition on MAPK signaling pathway.

Imiquimod-induced psoriasis mice model and TNF-α or IL-17A induced HaCAT cells, an experimental model in vitro for psoriasis [15], were constructed to explore the effect of BRD4 inhibition on psoriasis and the specific mechanism about MAPK pathway.

## Results

### BRD4 expression was upregulated in injured skin on the back of imiquimod-induced mice

**Fig. 1:**
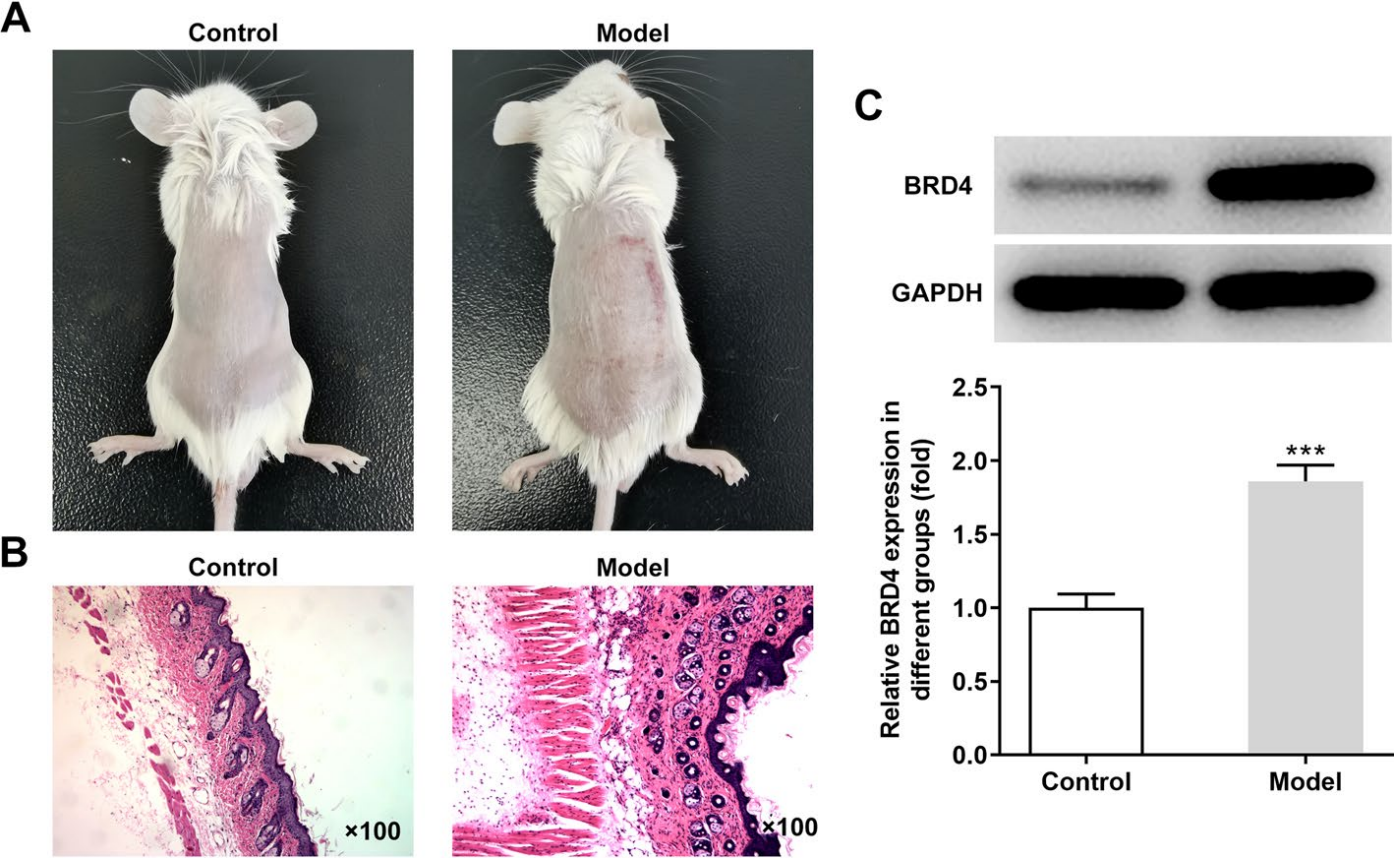
BRD4 expression was upregulated in injured skin on the back of imiquimod-induced mice. **A** Skin on the back of mice was photographed. **B** Pathological changes of skin on the back of mice was observed by HE staining. **C** BRD4 expression in skin tissues on the back of mice was detected by Western blot analysis. ***P < 0.001 vs. Control group

The skin was damaged on the back of mice induced by imiquimod for 7 days compared with control group (Fig. 1A). As shown in Fig. 1B, the dorsal epidermis of psoriasis mice was hyperplasia and thickened, mainly due to the increased number of spinous layer cells, accompanied by hyperplasia of dermal collagen fibers, red staining, and dense infiltration of dermal inflammatory cells. BRD4 expression was increased in injured skin tissues compared with control group (Fig. 1C).

### (+)-JQ1 weakened the skin injury on the back of imiquimod-induced mice

**Fig. 2:**
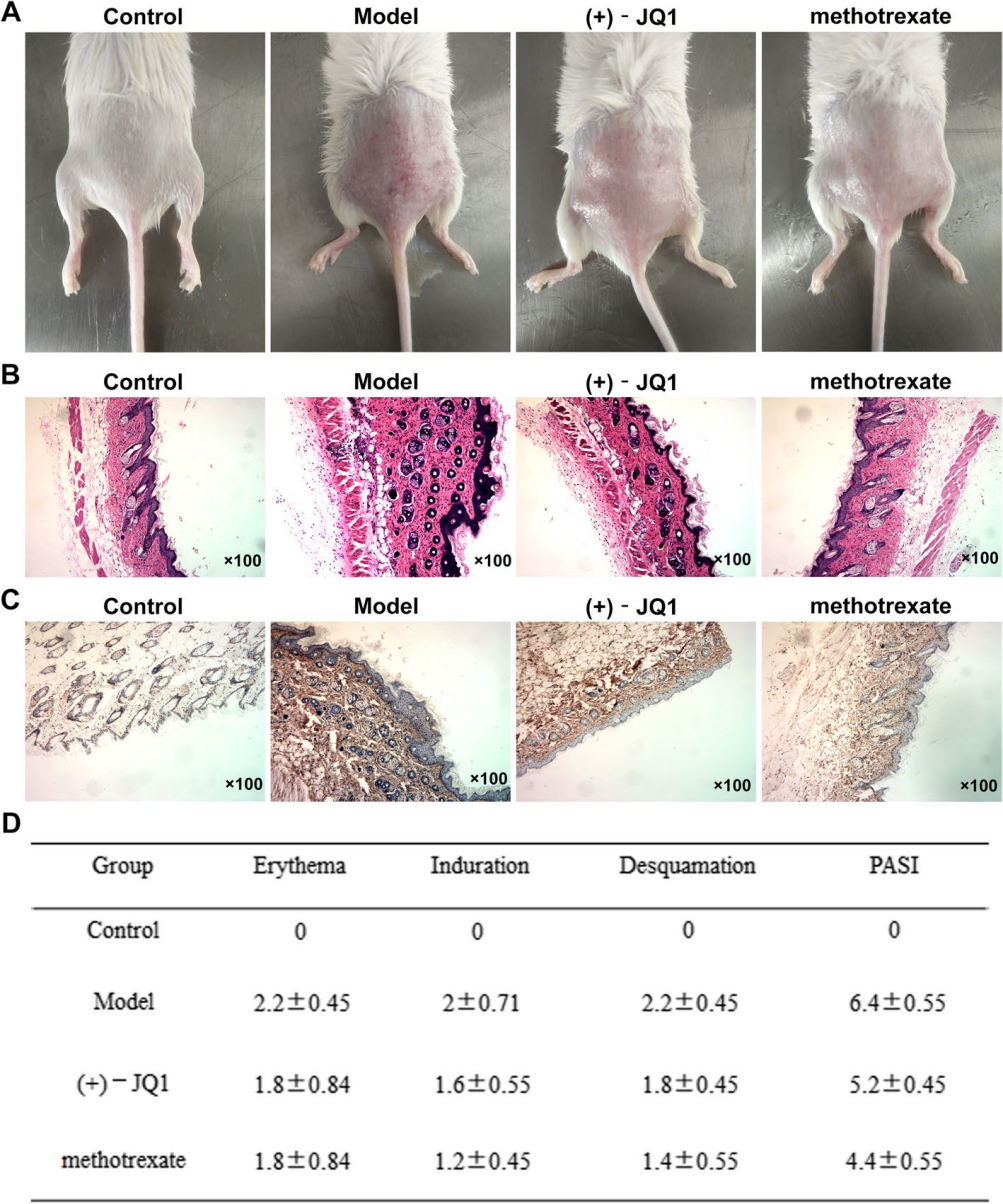
(+)-JQ1 weakened the skin injury on the back of imiquimod-induced mice. **A** Skin on the back of mice was photographed. **B** Pathological changes of skin on the back of mice was observed by HE staining. **C** KI67 expression in skin tissues on the back of mice was detected by Immunohistochemical analysis. **D** Degree of skin lesions in psoriasis was evaluated by PASI

The skin was seriously damaged on the back of mice induced by imiquimod for 7 days, and (+)-JQ1 or methotrexate could effectively alleviate the keratoderma injury on the back of imiquimod-induced mice (Fig. 2A). Compared with the Model group, the epidermis of the (+)-JQ1 group was significantly thinner, the collagen fibers of the dermis were sparse, and the inflammatory cells were less. The epidermis of methotrexate-treated mice was close to the histological changes of the epidermis of normal mice (Fig. 2B). KI67 expression was highly expressed in skin tissue samples of mice in Model group, which was decreased by the treatment of (+)-JQ1 or methotrexate (Fig. 2C). PASI score (6.4 ± 0.55) was the highest in Model group and (+)-JQ1 or methotrexate could reduce the PASI score (Fig. 2D).

### (+)-JQ1 decreased the release of inflammatory factors in serum and increased the apoptosis of keratinocytes of imiquimod-induced mice

**Fig. 3:**
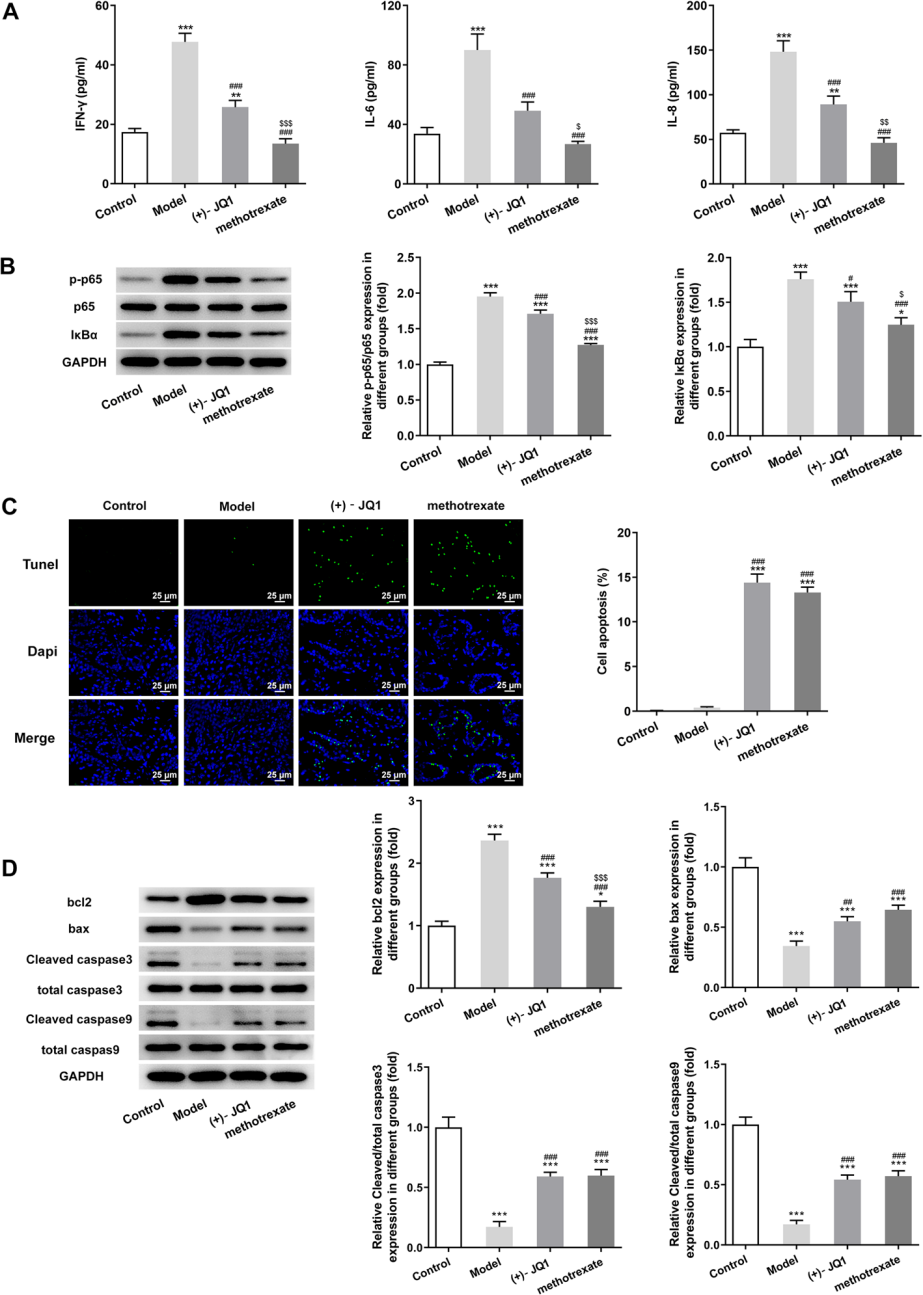
(+)-JQ1 decreased the release of inflammatory factors in serum and increased the apoptosis of keratinocytes of imiquimod-induced mice. **A** Expression levels of IFN-γ, IL-8 and IL-6 in serum were analyzed by ELISA assay. **B** Expression of p65 and IkBα in skin tissues was detected by Western blot analysis. **C** Apoptosis of keratinocytes in skin tissues was determined by TUNEL assay. **D** Expression of apoptosis-related proteins in skin tissues was detected by Western blot analysis. *P < 0.05, **P < 0.01 and ***P < 0.001 vs. Control group. ^#^*P* < 0.05, ^##^*P* < 0.01 and ^###^*P* < 0.001 vs. Model group. ^$^*P* < 0.05, ^$$^*P* < 0.01 and ^$$$^*P* < 0.001 vs. (+)-JQ1 group

The levels of inflammatory factors (IFN-γ, IL-8 and IL-6) in serum of imiquimod-induced rat were increased and (+)-JQ1 or methotrexate could suppress the inflammation (Fig. 3A). The expression of p-p65/p65 and IkBα in serum of imiquimod-induced rat was also upregulated, which was decreased by (+)-JQ1 or methotrexate (Fig. 3B). TUNEL assay indicated that apoptosis of keratinocytes was almost not happened in imiquimod-induced mice, which was enhanced by (+)-JQ1 or methotrexate (Fig. 3C). As shown in Fig. 3D, the bcl2 expression was increased, while the expression of bax, Cleaved caspase3/total caspase3 and Cleaved caspase9/total caspase9 was decreased in skin tissues of imiquimod-induced mice, which was partially reversed by (+)-JQ1 or methotrexate.

### (+)-JQ1 suppressed the MAPK signaling pathway in imiquimod-induced mice

**Fig. 4:**
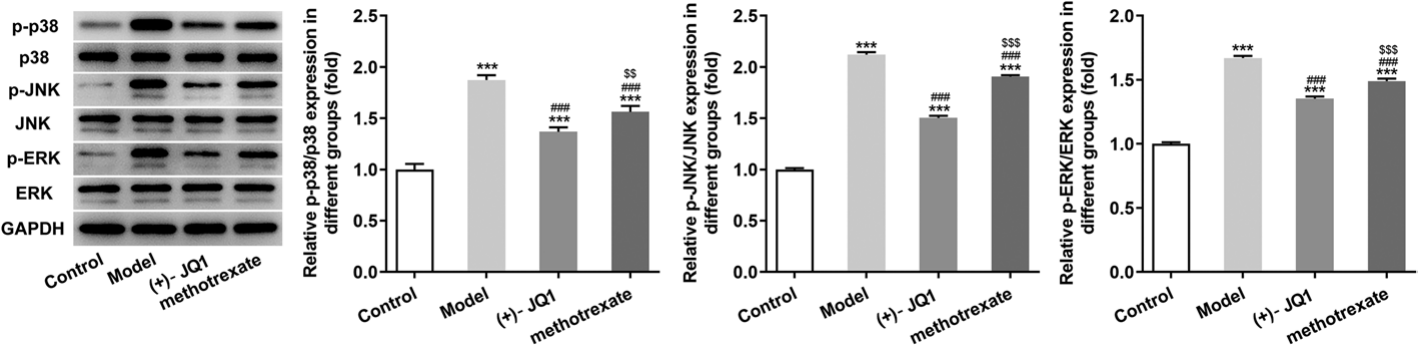
(+)-JQ1 suppressed the MAPK signaling pathway in imiquimod-induced mice. The expression of MAPK pathway-related proteins in skin tissues was detected by Western blot analysis. ***P < 0.001 vs. Control group. ^###^*P* < 0.001 vs. Model group. ^$$^*P* < 0.01 and ^$$$^*P* < 0.001 vs. (+)-JQ1 group

The expression of p-p38/p38, p-JNK/JNK and p-ERK/ERK in skin tissues of imiquimod-induced mice was increased, which was suppressed by the treatment of (+)-JQ1 or methotrexate (Fig. 4).

### BRD4 expression was increased in HaCAT cells induced by TNF-α or IL-17A

**Fig. 5:**
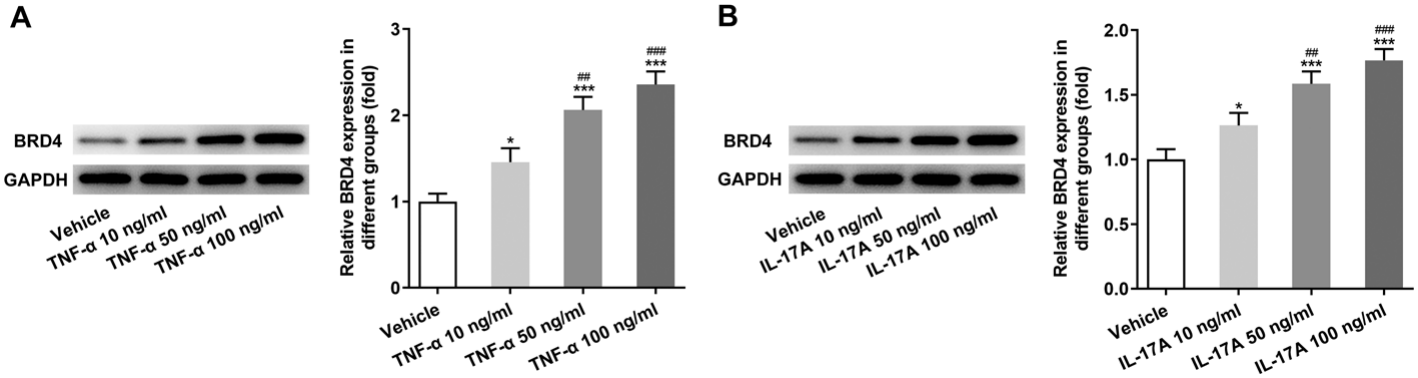
BRD4 expression was increased in HaCAT cells induced by TNF-α or IL-17A. **A** BRD4 expression in HaCAT cells induced by TNF-α was detected by Western blot analysis. *P < 0.05 and ***P < 0.001 vs. Vehicle group. ^##^*P* < 0.01 and ^###^*P* < 0.001 vs. TNF-α 10 ng/ml group. **B** BRD4 expression in HaCAT cells induced by IL-17A was detected by Western blot analysis. *P < 0.05 and ***P < 0.001 vs. Vehicle group. ^##^*P* < 0.01 and ^###^*P* < 0.001 vs. IL-17A 10 ng/ml group

After HaCAT cells induced by TNF-α, BRD4 expression was gradually increased from 10 to 50 ng/ml (Fig. 5A). Similarly, BRD4 expression was gradually increased in HaCAT cells treated with IL-17A from 10 ng/ml to 50 ng/ml (Fig. 5B). 100 ng/ml TNF-α or IL-17A has no obvious effect on BRD4 expression compared with 50 ng/ml TNF-α or IL-17A. Therefore, 50 ng/ml TNF-α or IL-17A was selected for subsequent experiment.

### (+)-JQ1 suppressed the viability of HaCAT cells induced by TNF-α or IL-17A

**Fig. 6:**
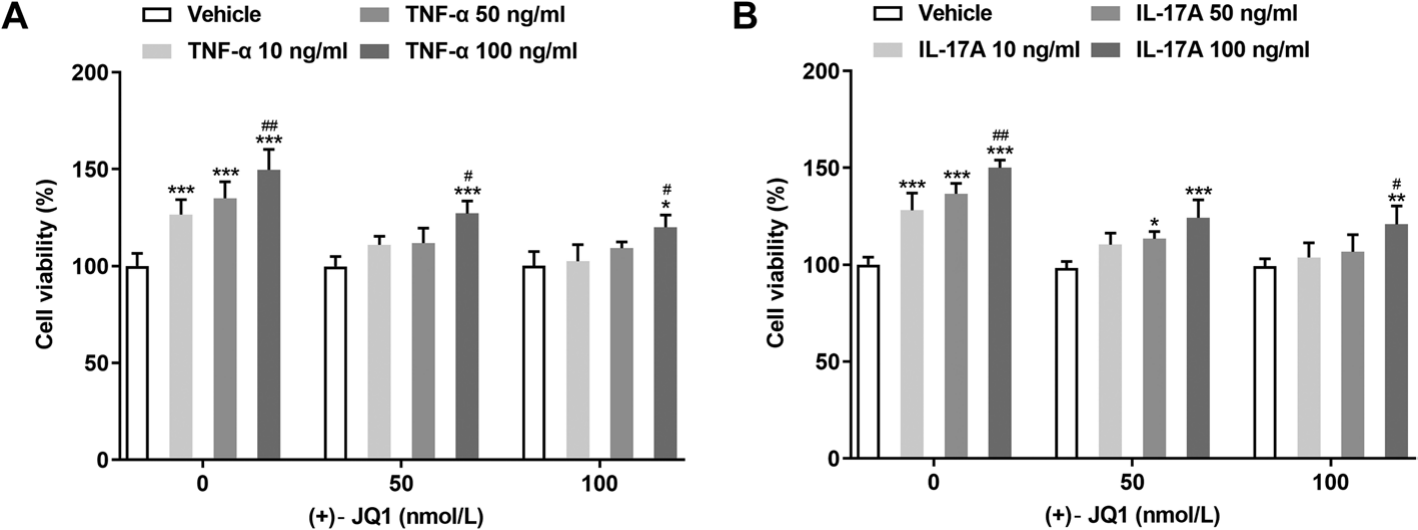
(+)-JQ1 suppressed the viability of HaCAT cells induced by TNF-α or IL-17A. **A** Viability of HaCAT cells induced by TNF-α was determined by CCK-8 assay. *P < 0.05 and ***P < 0.001 vs. Vehicle group. ^#^*P* < 0.05 and ^##^*P* < 0.01 vs. TNF-α 10 ng/ml group. **B** Viability of HaCAT cells induced by IL-17A was determined by CCK-8 assay. *P < 0.05, **P < 0.01 and ***P < 0.001 vs. Vehicle group. ^#^*P* < 0.05 and ^##^*P* < 0.01 vs. IL-17A 10 ng/ml group

The viability of HaCAT cells induced by TNF-α or IL-17A was increased and (+)-JQ1 suppressed the viability of the TNF-α or IL-17A induced HaCAT cells from 50 to 100 nM (Fig. 6A, B). Therefore, 100 nM (+)-JQ1 was chosen for next experiment.

### (+)-JQ1 suppressed the inflammation and proliferation, and promoted the apoptosis of HaCAT cells induced by TNF-α or IL-17A

**Fig. 7:**
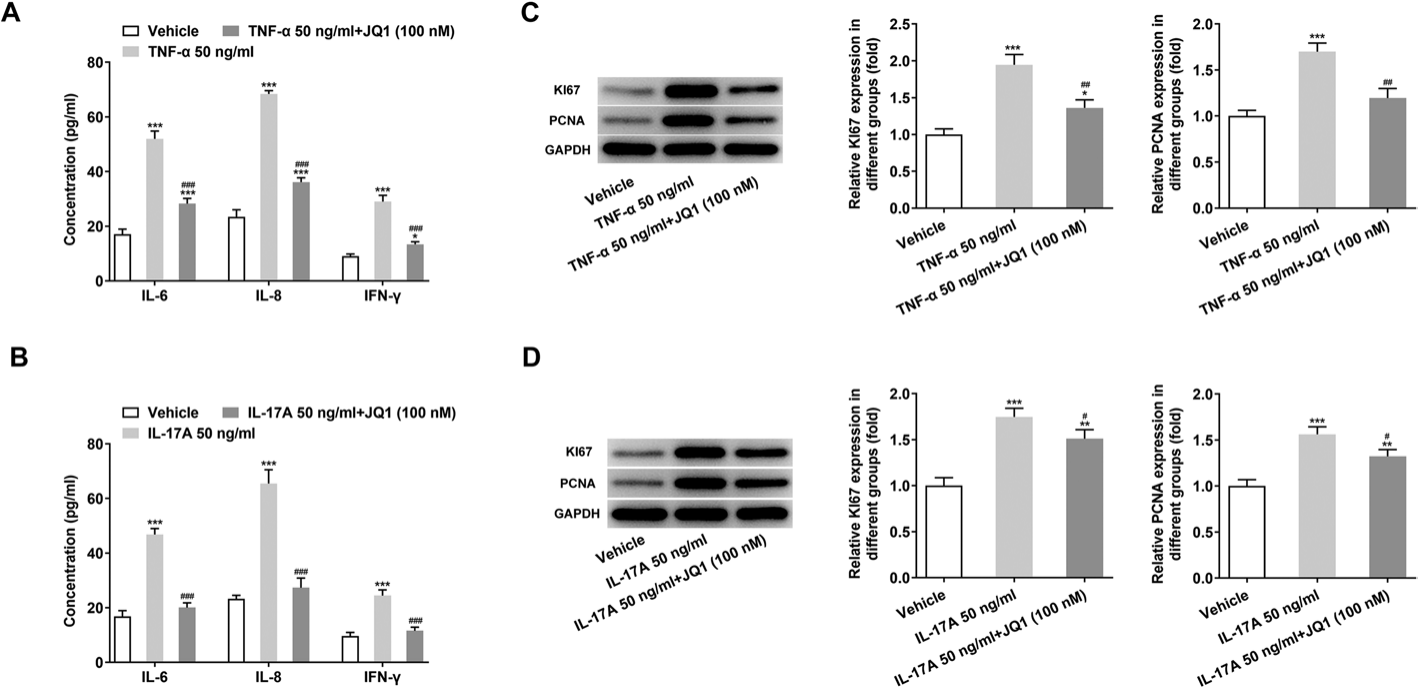
(+)-JQ1 suppressed the inflammation and proliferation, and promoted the apoptosis of HaCAT cells induced by TNF-α or IL-17A. **A** Expression levels of IFN-γ, IL-8 and IL-6 in culture supernatant of HaCAT cells induced by TNF-α were analyzed by ELISA assay. *P < 0.05 and ***P < 0.001 vs. Vehicle group. ^###^*P* < 0.001 vs. TNF-α 50 ng/ml group. **B** Expression levels of IFN-γ, IL-8 and IL-6 in culture supernatant of HaCAT cells induced by IL-17A were analyzed by ELISA assay. ***P < 0.001 vs. Vehicle group. ^###^*P* < 0.001 vs. IL-17A 50 ng/ml group. **C** Expression of KI67 and PCNA in HaCAT cells induced by TNF-α was detected by Western blot analysis. *P < 0.05 and ***P < 0.001 vs. Vehicle group. ^##^*P* < 0.01 vs. TNF-α 50 ng/ml group. **D** Expression of KI67 and PCNA in HaCAT cells induced by IL-17A was detected by Western blot analysis. **P < 0.01 and ***P < 0.001 vs. Vehicle group. ^#^*P* < 0.05 vs. IL-17A 50 ng/ml group

**Fig. 8:**
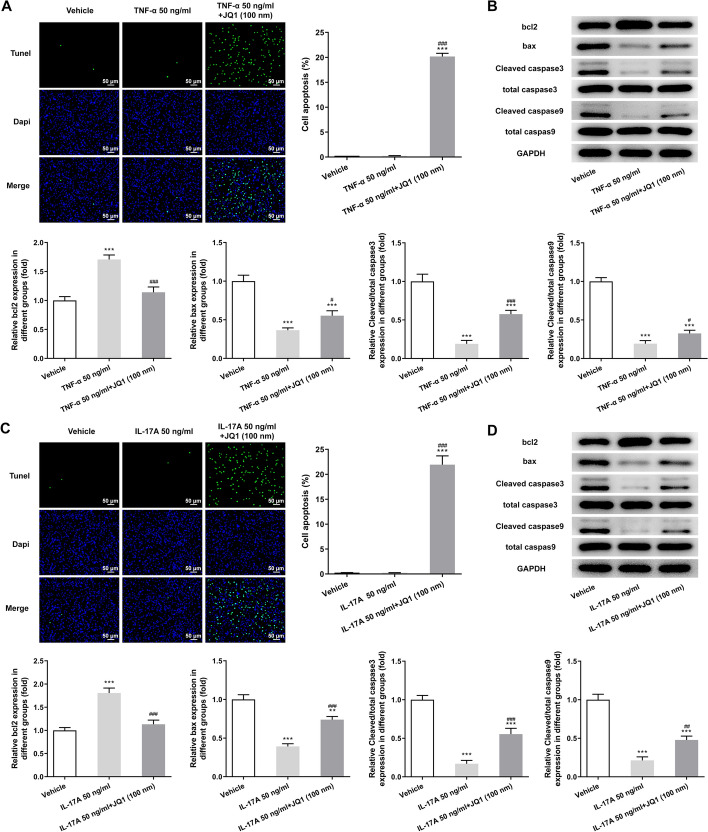
(+)-JQ1 promoted the apoptosis of HaCAT cells induced by TNF-α or IL-17A. **A** Apoptosis of HaCAT cells induced by TNF-α was determined by TUNEL assay. **B** Expression of apoptosis-related proteins in HaCAT cells induced by TNF-α was detected by Western blot analysis. *P < 0.05, **P < 0.01 and ***P < 0.001 vs. Vehicle group. ^#^*P* < 0.05 and ^###^*P* < 0.001 vs. TNF-α 50 ng/ml group. **C** Apoptosis of HaCAT cells induced by IL-17A was determined by TUNEL assay. **D** Expression of apoptosis-related proteins in HaCAT cells induced by IL-17A was detected by Western blot analysis. **P < 0.01 and ***P < 0.001 vs. Vehicle group. ^##^*P* < 0.01 and ^###^*P* < 0.001 vs. IL-17A 50 ng/ml group

The levels of IFN-γ, IL-8 and IL-6 in culture supernatant of TNF-α or IL-17A (50 ng/ml) induced HaCAT cells were elevated, which was downregulated by 100 nM (+)-JQ1 (Fig. 7A, B). The expression of KI67 and PCNA in TNF-α or IL-17A (50 ng/ml) induced HaCAT cells was also increased, which was decreased by 100 nM (+)-JQ1 (Fig. 7C, D). The apoptosis of HaCAT cells induced by TNF-α or IL-17A was promoted by the treatment of 100 nM (+)-JQ1 (Fig. 8A, C). The bcl2 expression was increased, while the expression of bax, Cleaved caspase3/total caspase3 and Cleaved caspase9/total caspase9 was decreased in TNF-α or IL-17A (50 ng/ml) induced HaCAT cells, which was partially reversed by 100 nM (+)-JQ1 (Fig. 8B, D).

### (+)-JQ1 suppressed the MAPK signaling pathway in HaCAT cells induced by TNF-α or IL-17A

**Fig. 9:**
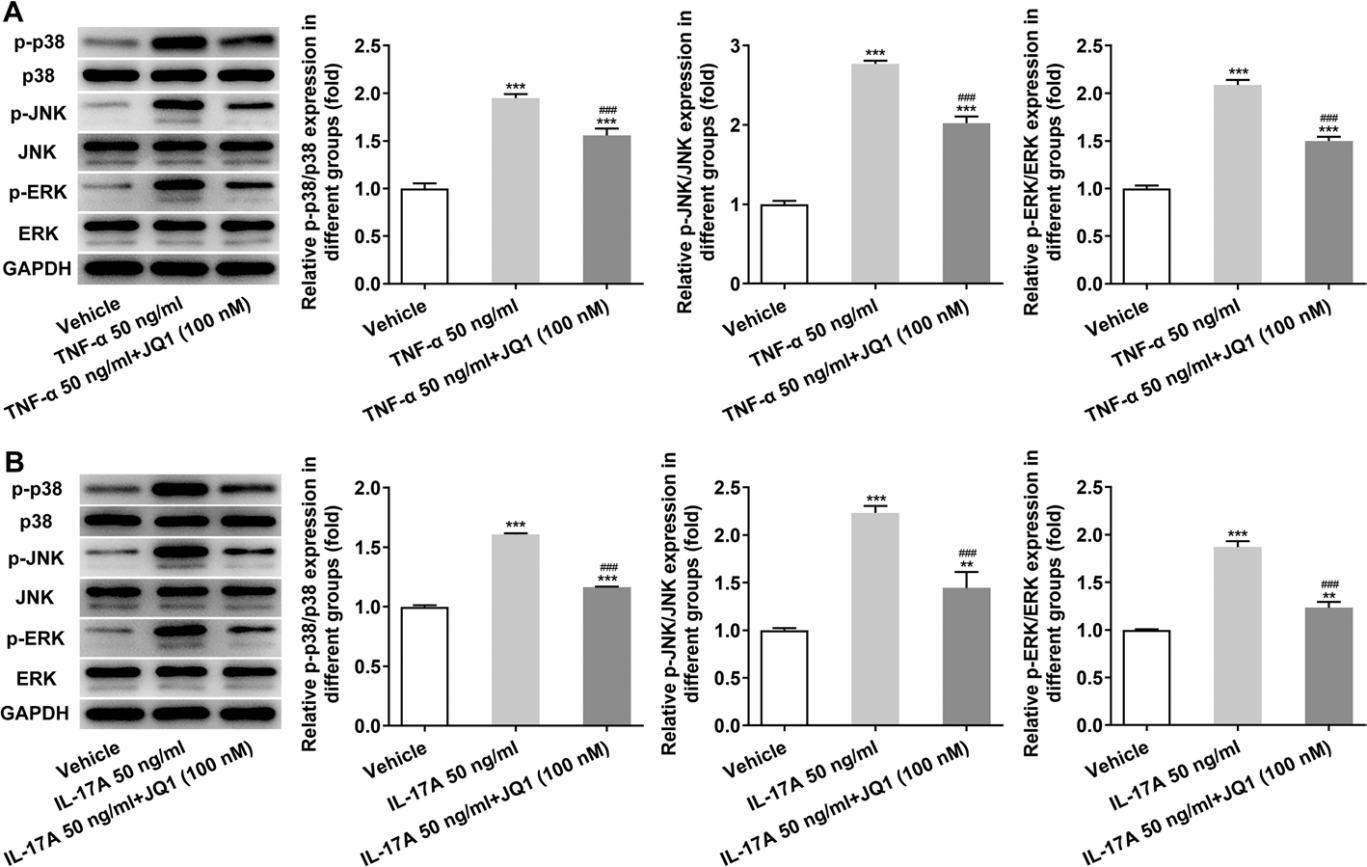
(+)-JQ1 suppressed the MAPK signaling pathway in HaCAT cells induced by TNF-α or IL-17A. **A** Expression of MAPK pathway-related proteins in HaCAT cells induced by TNF-α was detected by Western blot analysis. ***P < 0.001 vs. Vehicle group. ^###^*P* < 0.001 vs. TNF-α 50 ng/ml group. **B** Expression of MAPK pathway-related proteins in HaCAT cells induced by IL-17A was detected by Western blot analysis. **P < 0.01 and ***P < 0.001 vs. Vehicle group. ^###^*P* < 0.001 vs. IL-17A 50 ng/ml group

The expression of p-p38/p38, p-JNK/JNK and p-ERK/ERK in TNF-α or IL-17A (50 ng/ml) induced HaCAT cells was increased, which was suppressed by the treatment of 100 nM (+)-JQ1 (Fig. 9A, B).

## Discussion

The keratinocytes have receptors for most psoriasis-associated cytokines and chemokines, they represent tissue cells that respond critically to the psoriatic microenvironment. The inflammatory response is accelerated by the production of other cytokines in response to psoriatic cytokines [16–18]. Activated keratinocytes are involved in maintaining and amplifying skin inflammation by releasing inflammatory cytokines and chemokines, which are critical for recruitment of T cells, neutrophils and inflammatory myeloid dendritic cells [19–21]. In the present study, we found that inflammatory factors (IFN-γ, IL-8 and IL-6) were increased in serum of imiquimod-induced mice and culture supernatant of HaCAT cells induced by TNF-α or IL-17A.

As a classical cell signal transduction pathway, MAPK/NF-κB signaling pathway plays an important role in inducing the expression and aggregation of pro-inflammatory factors and anti-inflammatory factors, as well as activating and regulating inflammatory response in psoriasis [22, 23]. Here, the expression of phosphorylation of NF-κB (p-p65, IkBα), MAPK (p-p38, p-JNK and p-ERK) was also activated in skin tissues of imiquimod-induced mice and HaCAT cells induced by TNF-α or IL-17A. MAPK pathway can also regulate the proliferation and differentiation of keratinocytes. Previous study has shown that MAPK pathway can promote the expression of KI67, PCNA and keratin in keratinocytes, so as to promote the proliferation of keratinocytes [24]. This study indicated that KI67 expression was increased in skin samples of imiquimod-induced mice and the expression of KI67 and PCNA was all increased in HaCAT cells induced by TNF-α or IL-17A.

Zhu et al. found that the blockage of BRD4 could obviously alleviate the inflammation and oxidative stress in aortic banding-operated mice [25]. Downregulation of BRD4 decreased apoptosis and inflammation in BEAS-2B cells induced by cigarette smoke extract [26]. BRD4 could increase transcriptional transactivation activity and stability of NF-κB in the nucleus [27]. BRD4 was necessary for coupling NF-κB to express inflammatory genes, and knockdown of BRD4 alleviated exuberant virus-induced mucosal airway inflammation [28]. Inhibition of BRD4 has been demonstrated to suppress the MAPK signaling pathway in spinal cord ischemia reperfusion injury and diabetic intervertebral disc degeneration [11, 14]. Our study showed that BRD4 expression was increased in skin samples of imiquimod-induced mice and HaCAT cells induced by TNF-α or IL-17A. In addition, inhibition of BRD4 decreased the expression levels of inflammatory factors and proliferation-related proteins while increased the expression of apoptosis-related proteins by suppressing the MAPK signaling pathway.

In conclusion, the findings from our study suggest that BRD4 is functioned in regulating the proliferation and inflammation of keratinocytes through MAPK pathway in psoriasis. Inhibition of BRD4 may possess therapeutic efficacy in the treatment of psoriasis.

## Materials and methods

### Imiquimod-induced psoriasis mice model

Male BALB/c mice aged 6 weeks ate standard animal feed and drink filtered tap water freely for 7 days in a controlled room with the temperature between 22 to 26 °C and humidity between 40 to 70%. Mice were used to shave their dorsal skins, and imiquimod cream was applied locally once a day for 7 consecutive days to induce psoriasis-like dermatitis. (+)-JQ1 (50 mg/kg) was given intraperitoneal injection on the 7th day, once a day, totally for 13 days. The mice were sacrificed on the 20th day and skin tissue samples were obtained from the diseased areas. 25% of the skin was stored with 4% paraformaldehyde at room temperature, while the rest of the skin was stored at − 80 °C. The blood samples were obtained from abdominal aortas and centrifuged at 3000 r/min for 10 min to obtain the serum samples which were stored at − 80 °C. Experimental groups were randomly divided into Control group, Model group, (+)-JQ1 group and methotrexate group (*n* = 5). The animal experiment was approved and supervised by the Animal Care and Use Committee and the Animal Ethics Committee at Shandong Provincial Qianfoshan Hospital, Cheeloo College of Medicine, Shandong University.

### Haematoxylin–eosin staining

Paraffin sections of skin tissues were stained by hematoxylin and eosin (H&E; Beyotime), and pathological skin changes were observed under a light microscope (Olympus Corporation; magnification, 100×).

### Psoriasis area and severity index (PASI)

PASI score was adopted for the degree of the three indexes (Erythema, Induration and Desquamation) at the skin lesions of mice, and the sum of the scores of the three indexes was the total score. PASI scoring criteria: none (0): no erythema scales on the surface; mild (1): some lesions were covered with scales, mainly fine scales, slightly higher than the normal skin surface, reddish; moderate (2): most lesions were completely or incompletely covered with scales, scales were patchy, moderately raised, the edge of the plaque was round or sloping, red; severe (3): almost all lesions covered with scales, scales thick layer, thick lesions, prominent protuberance, crimson; extremely severe (4): all lesions. The total score was the sum of the three indexes (0 ~ 12 points).

### Immunohistochemical analysis

The paraffin sections were dewaxened, hydrated, sealed, and cold-repaired with antigen repair solution. The sections were incubated with KI67 antibody (Abcam) at 4 °C overnight. After placed at room temperature for 30 min the next day, the sections were first added with reaction enhanced solution for 10 min, followed by the treatment with secondary antibody for 20 min at room temperature. Then, sections were colored with the treatment of DAB for 5–8 min, counterstained with hematoxylin for 20 s, dehydrated and sealed. Finally, KI67 expression was observed under a light microscope (Olympus Corporation; magnification, 100×).

### Cell culture and cell induction

Human immortal keratinocyte line (HaCAT) was provided from CoBioer (Nanjing, China). HaCAT cells were cultured in Dulbecco’s modified Eagle's medium (DMEM) containing 10% fetal bovine serum (FBS; Thermo Fisher Scientific, Inc.) at 37 °C of 5% CO_2_.

HaCAT cells were, respectively, induced by TNF-α or IL-17A at concentrations of 10 ng/ml, 50 ng/ml and 100 ng/ml for 24 h to construct the vitro model of psoriasis.

### ELISA assay

The serum was dissolved at room temperature and mixed well. The cell culture supernatant of HaCAT cells after indicated treatment was obtained after centrifugation (1000 r/min, 10 min). The levels of IFN-γ, IL-8 and IL-6 in the mice serum were detected by Mouse IFN-γ ELISA Kit (Beyotime), Mouse IL-8 ELISA Kit (Wuhan Saipei Biotechnology Co., Ltd) and Mouse IL-6 ELISA Kit (Beyotime) and the levels of IFN-γ, IL-8 and IL-6 in the culture supernatant of HaCAT cells were detected by Human ELISA Kits (Beyotime).

### TUNEL assay

Keratinocyte apoptosis at the back of mice skin was detected by terminal deoxynucleotidyl transferase dUTP nick end labeling (TUNEL) assay in accordance with the manufacturer protocol (Roche, USA). After washed with PBS for three times, skin sections were incubated with DAPI for 10 min, which were then observed and photographed using a fluorescence microscope in a mounting medium (Olympus Corporation; magnification, 400×).

### Western blot analysis

Total protein lysates in the rest of frozen skin samples and HaCAT cells after indicated treatment were obtained with RIPA buffer. A bicinchoninic acid (BCA) protein assay kit (Beyotime) was applied to detect protein concentration. 50 µg proteins were separated on 12% gel with sodium dodecyl sulfate polyacrylamide gel electrophoresis (SDS-PAGE) and transferred to a polyvinylidene fluoride (PVDF) membrane. Blocked with 5% milk for 2 h at room temperature, the membrane was incubated with the primary antibodies of BRD4, p-p65, IkBα, p65, bcl2, bax, cleaved caspase3, cleaved caspase9, total caspase3, total caspase9, p-P38, p-JNK, p-ERK, P38, JNK, ERK, KI67, PCNA and GAPDH at 4 °C overnight. After washing of tris-buffered saline with Tween (TBST) for three times, the membranes were incubated with the HRP-labeled secondary antibody at room temperature for 1 h. The protein bands were detected by enhanced chemiluminescence (ECL; Millipore, USA) and quantified using Image-Pro Plus software (version 6.0; Media Cybernetics, Inc.).

### CCK-8 assay

HaCAT cells were seeded at 5 × 10^3^ cells/well in the 96-well plate and cultured for overnight at 37 °C. HaCAT cells were treated with TNF-α or IL-17A (10 ng/ml, 50 ng/ml and 100 ng/ml) adding or not adding (+)-JQ1 (50 nmol/l and 100 nmol/l) for 24 h. 10 μl CCK-8 solution (Beyotime) was added to each well of 96-well plate which was incubated at 37 °C for 1 h. The optical density (OD) value at 450 nm was determined with an enzyme-linked immunosorbent assay reader. (+)-JQ1 is a BRD4 inhibitor.

### Statistical analysis

Statistical analysis software Graphpad8.0 was used, experimental data were represented by mean ± standard deviation (SD). Differences between the two groups were calculated by Student-*t* test. One-way ANOVA with Tukey's post hoc test was used for the comparison of mean values when more than two groups. Differences were statistically significant when *P* < 0.05.

## Data Availability

The experimental data will be available on the request.
